# Human Injuries Associated with the Transport of Horses by Road

**DOI:** 10.3390/ani13101594

**Published:** 2023-05-10

**Authors:** Christopher B. Riley, Barbara Padalino, Chris W. Rogers, Kirrilly R. Thompson

**Affiliations:** 1 Department of Clinical Studies, Ontario Veterinary College, University of Guelph, Guelph, ON N1G 2W1, Canada; 2 School of Veterinary Science, Massey University, Palmerston North 4442, New Zealand; c.w.rogers@massey.ac.nz; 3 Dipartimento di Scienze e Tecnologie Agro-Alimentari, Alma Mater Studiorum—Università di Bologna, Viale Fanin 50, 40126 Bologna, Italy; barbara.padalino@unibo.it; 4 School of Medicine and Public Health, University of Newcastle, Newcastle, NSW 2300, Australia; kirrilly.thompson@newcastle.edu.au; 5 Hunter New England Local Health District, Wallsend, NSW 2287, Australia

**Keywords:** human, injury, equine, horse, transport, road, transportation, risk, one welfare

## Abstract

**Simple Summary:**

There is an increased understanding of shared human–animal risk in terms of “one welfare”, whereby when animals are at risk, so are people. Reducing the risk and preventing injury to one species may also prevent injury to the other. The authors of the current manuscript considered this approach to study road equine transport-related injuries to humans in New Zealand and so aimed to determine their frequency and associated factors. New Zealand horse industry participants were surveyed on their horse industry, activities, and road transport experiences and asked if they had experienced horse-related self-injury. There were 112/1067 (10.5%) owners and carers injured while preparing, loading, traveling, or unloading. Of these, four in ten had multiple injury types, and a third had several body regions affected. Hand injury was most common, followed by the foot, arm, head, or face. Injuries were associated with the responder’s industry educational background, driving experience, and reporting a horse injured during road transport in the past two years. Findings support wearing helmets and gloves, and adopting strategies designed to eliminate equine injuries associated with the road transport of horses to reduce the risk of injury to their handlers.

**Abstract:**

There is an increased understanding of shared human–animal risk in terms of “one welfare”, whereby when animals are at risk, so are people, so preventing injury to one species may also prevent injury to the other. Because transport-related injuries to horses are common, the authors considered this paradigm to study road equine transport-related injuries to humans in New Zealand. The aim was to determine their frequency and associated factors by distributing a survey to horse industry participants through industry organisations asking about their horse activities, road transport experiences, and any related self-injury. There were 112/1067 (10.5%) handlers injured while preparing (13/112), loading (39/112), traveling (6/112), or unloading (33/112). Of these, 40% had multiple injury types, and 33% had several body regions affected. Hand injuries were most common (46%), followed by the foot (25%), arm (17%), and head or face (15%). Median recovery time was 7 days. Injuries were associated with the responder’s industry educational background, years of driving experience, and reporting a horse injured during road transport in the past two years. Wearing helmets and gloves, and adopting strategies designed to eliminate equine injuries associated with the road transport of horses to reduce the risk of injury to their handlers are recommended.

## 1. Introduction

There is a resurgence in interest in the risk and prevention of human injury associated with equestrian activities [1,2]. This is partly because, despite advances in the design and use of safety equipment for participants in equine activities, the frequency of human–horse-related injuries has not declined [3,4,5]. Furthermore, equine-related injury is now recognized as a serious occupational risk in related industry sectors and farming [2,5,6]. The approaches to its study have moved from merely descriptive [7] to more in-depth analyses of associative factors [5,8]. Most of these studies focus on demographic or specific medical assessment and treatment outcomes with little reference to the human–animal bond and its influences [9]. However, there is an increasing understanding of the paradigm of shared human–animal risk. This concept examines injury risk in terms of “one welfare”, whereby when animals are at risk, so are people [10,11,12,13]. The corollary is that reducing the risk and preventing injury to one species may also prevent injury to the other [14]. Based on this reflection, the authors of the current manuscript hypothesized that such an approach might be taken in studying road-transport-related injuries to humans and horses in New Zealand. 

In New Zealand, the equine population is mainly located near the country’s major human population centres, but locations for equestrian, breeding, and racing activities are distributed at significant distances throughout the country, necessitating the frequent transport of horses by road [15,16,17]. A recent equine road transport survey by the authors confirmed that this routine industry activity places these horses at significant risk of injury and compromised welfare [18,19]. In this study of New Zealand’s amateur and professional equine industry participants, 17.7% of horse carers reported an incident resulting in death or injury to their horses during the previous two-year period, and 22.2% described transport-related behavioural problems. Similar problems have been reported for Australian, Italian, and United Kingdom horse populations [20,21,22,23,24]. Whereas some studies have reported predominantly in-transit horse injuries [23], others have reported these problems in all phases of transport, including preloading, loading, and unloading [19,20,21,22,24]. 

Few recent publications have investigated the interacting factors of motor vehicles, horses, and people leading to human injury [8,24,25,26]. Most of these studies have focused on incidents involving loose or ridden horses or horse-drawn vehicles that were struck by or experienced a near-miss with motor vehicles on roads. Such events may result in severe injury or fatality to riders, handlers, and drivers, attracting great public attention and outrage. However, transporting horses by road is likely to be a more frequent occurrence. The frequency of exposure to injury risk associated with this routine management activity is high for horse industry workers, owners, and caregivers [4,27]. Human injury associated with the loading of horses has been reported anecdotally [28], but only two studies have purposefully investigated human injury in association with the road transport of horses within surveys of equine-transport-related injuries [24,29]. It has been suggested that by observing and recording human and equine behaviour simultaneously when transporting horses, issues of animal welfare and human safety can be concurrently addressed [29]. To that end, as part of a larger study of equine transport in New Zealand, the authors of the current manuscript surveyed horse owners and carers using equine road transport, simultaneously investigating equine-transport-related behavioural problems and injuries and human factors associated with these welfare concerns [18,19,30,31]. At the same time, respondents were surveyed regarding injuries they had personally sustained in association with the road transport of horses in New Zealand. For this aspect of the work reported in the current article, the authors aimed to determine the frequency with which human injuries related to the road transport of horses were sustained (if any) and to explore possible associations with the responders’ demographics and experience, vehicle and journey frequency, equine injury, and transport-related behavioural problems.

## 2. Materials and Methods

### 2.1. Respondents

The target population for the survey was horse industry participants (professional and amateur) living within New Zealand and involved in transporting horses by road. Eligible voluntary survey participants must have had at least one horse in their care and have organized or provided transportation for one or more horses during the two years before completion of the survey. The target population was estimated at 90,000 New Zealand equine industry participants [32,33]; 1055 completed surveys were required to obtain a 95% confidence interval with an error level of ±3%. 

### 2.2. Survey

The survey was designed using an iterative review process to ensure valid questionnaire results [34,35], using commercial software (Qualtrics, New Zealand) to facilitate the capture of data on a range of factors associated with the transport of horses by road in New Zealand, including human and equine injury and horse behavioural problems [18,19,30,31]. A mixture of open-ended and closed-ended questions sought information from respondents concerning their demographic details, involvement with sectors within the New Zealand equine industry, the specific type of activities in which they participated, their related experience, and transport-related practices, horse, and road journey details. Survey respondents were also asked to answer questions describing the most recent transport-related injury (if any) to a horse for which they were responsible and/or to themselves (i.e., human injury) during the two years before they completed the survey. For comparison with those injured, those who did not have a horse injured during this period were asked questions about their most recent horse road transport experience. The authors published the survey questionnaire, available as supplementary material with the cited references [18,30]. The questions that were specific to human injury are listed in the supplementary materials (Table S1). The study protocol was registered with the Massey University Human Ethics Committee as low risk (Ethics Notification Number: 4000017178). 

New Zealand professional and amateur horse organizations and their members were asked to distribute invitations to participate, including the online survey link. A list of the organisations contacted by the research team was published [30]. Contact with the industry officials initially occurred by telephone. They were recontacted with a second request six weeks after the survey link was activated. As described previously, industry organization staff who elected to participate forwarded the survey link to members and others via email, the internet, and other social networking sites, such as Facebook, with the engagement of others by supportive individuals on social media [36]. The survey link was open for participation for 12 weeks. 

### 2.3. Data Analysis

Analyses of equine injuries and problem behaviours associated with this survey regarding the road transport of horses in New Zealand have been published previously [18,19,30,31]. In the present article, the authors focus on the equine-transport-related risk of human (owners and handlers) injury. Variable frequencies and summative statistics were calculated in Excel (Microsoft Excel, Version 16.46, 2021, Microsoft Corporation, Redmond, WA, USA). The median and interquartile ranges (IQR = third quartile minus first quartile) were calculated for those data associated with variables not normally distributed. The mean and standard deviation were calculated for continuous variables that were normally distributed. Statistical analyses were performed using R version 4.2.2.2 (GUI 1.79 Big Sur ARM build, R Foundation for Statistical Computing, Vienna, Austria). Logistic regression models were built using the “glm” function from the base “stats” package in R, with human injury (yes/no) as the binary response variable. As most human injuries reported did not occur at the same time as the reported horse injury, univariate logistic regression was limited to those variables associated with the responder and their experience and whether they indicated that a horse was injured or was recorded as having a transport-related behavioural problem during the two years before completing the survey. Statistical significance was determined (*p* values) using the Wald test. Variables with a *p* < 0.20 were considered for investigation in a multivariable model for human injury. Variables were eliminated using a stepwise backward elimination procedure until all variables in the final model had *p* < 0.05. Outcomes of analyses are reported as coefficients, standard error, and odds ratios (OR) with 95% confidence intervals for the OR of each variable.

## 3. Results

### 3.1. Survey Respondents

There were 1133 valid responses to the New Zealand survey of equine road transport. Of these, 1067 (94.2%) answered questions about their experience of human (self) injury associated with the road transport of horses. This provided a sample size representative of the study population with a 95% confidence level and an error level of ±3%. The frequency table for variables explored for their possible associations with human injuries sustained while transporting horses by road in New Zealand is available in the supplementary materials (Table S2). The medians and interquartile ranges for the age of injured, uninjured, and all respondents were 37 (IQR = 29), 46 (IQR = 22), and 45 (IQR = 24) years, respectively. The medians and IQRs for the driving experience of injured, uninjured, and all respondents were 12 (IQR = 28), 25 (IQR = 25), and 24 (IQR = 26) years, respectively. The medians and IQRs for the horse handling experience of injured, uninjured, and all respondents were 21 (IQR = 28), 30 (IQR = 23), and 30 (IQR = 24) years, respectively. Further data and a description of other demographic information on the responding population were published [30].

### 3.2. Descriptive Data for Road Transport-Related Horse Injuries

One hundred and twelve respondents (112/1067; 10.5%) reported that they had sustained an injury associated with transporting one or more horses. Of these, only 16/112 (14.3%) occurred at the same time as a horse was injured during road transport; the remainder were not associated with a concurrent injury to a horse they were transporting. Of these 16 respondents, 7/16 sustained an injury at the same time as the injured horse they reported in the survey, and 9/16 occurred with a different horse. Most injuries were sustained while loading (39/112; 34.8%) or unloading (33/112; 29.5%), with fewer reported as occurring during preloading (13/112; 11.6%) or while in transit (6/112; 5.4%). The remainder of respondents (21/112; 18.8%) did not indicate when their injury was sustained while transporting a horse.

Seventy-three (73/112; 65.2%) injured respondents responded to additional questions regarding the nature and extent of their injuries, treatment, and recovery. Of these, 27/73 (40.0%) reported multiple concurrent injury types (Table 1), and 24/73 (32.9%) reported multiple body regions affected (Table 2). The most commonly injured body part was the hand (45.8%), followed in decreasing frequency by the foot (25%), arm (16.7%), head or face (15.3%), leg (12.5%), back (9.7%), chest (8.3%), stomach or abdomen (5.6%), neck (4.2%), and pelvis (2.8%) (Table 2).

Most respondents (30/72; 41.7%) reported self-treatment of their injuries, 15/72 (20.8%) sought medical assistance, 5/72 received first aid, and 3/72 (4.2%) were managed with physiotherapy or chiropractic treatment. The remainder (15/72; 20.8%) of responders did not treat their injuries. Of the injured respondents, 68/72 (94.4%) had recovered at the time of responding to the survey; 4/72 (5.6%) had not. For those that reported recovery, the time to recovery ranged from 0 to 365 days with a median of 7 days (IQR 26.5 days).

### 3.3. Variables Associated with Human Injury

In the univariate regression, respondent sex, age, years of driving experience, horse industry sector involvement, the highest horse qualification achieved, the vehicle usually used for horse transport, the frequency of horse transport, and reporting a transport-related horse injury in the survey were all found to have a significant association (*p* < 0.05) with equine-transport-related human injury (Table 3). The results of the multivariate logistic regression analyses are listed in Table 4 (χ^2^ = 46.7, df = 7, *p* < 0.001). Respondents’ horse industry qualifications, years of driving experience, and reporting a transport-related injury in the survey were associated with increased, decreased, or increased odds of human injury, respectively.

## 4. Discussion

The findings from the survey confirm that New Zealanders who transport horses are at a concerning risk of injury, estimated at 10.5% in the period covered by the survey. This compares to a recent study of Italian industry respondents to an equine transport survey that found 7.4% (10/140) reported a related injury that occurred during the two years before completing the survey [24]. An earlier Swedish study of loading horses into trailers found that 12% (11/95) of surveyed owners reported they had an associated injury at some time in the past (i.e., there was no defined retrospective period given) [29], and the respondent sample size surveyed (n = 99) was not representative of the estimated 500,000 Swedish horse industry participants [37]. Concurrent human and equine-transport-related injuries occurred in only 14% of injured respondents, compared with 40% (4/10) in the Italian study [24]. The Swedish researchers reported that 5% of respondents described equine and human injuries but did not indicate if these had occurred concurrently [29]. The proportion of concurrent injuries in the present and Italian studies [24] is modest but concerning and underscores the shared risk associated with the frequent event of horse transport. Although few respondents reported an injury that was concurrent with the equine incident they described with the same survey, the strong association found for those reporting both within the two-year timeframe merits further investigation of the human factors and behaviors that underpin this shared risk to welfare and the specific measures required for mitigation [1,38,39].

Transport-related injuries to horses occur in all phases of the activity: preloading, loading, in transit, and unloading [19,21,24]. This held true for human injury in the present study and an Italian study [24]. In common with the latter study, most injuries in the present study occurred during loading or unloading. These results confirm that the loading and unloading of horses pose a risk to human health that requires specific risk-reduction measures [29]. All equine-related journeys include these activities; therefore, studies that only investigate in-transit injuries to horse owners and carers are likely to provide inaccurate estimates of risk within the population studied. 

Descriptions of anatomic patterns and the severity of human injuries associated with horses are common but usually focus on those sustained by riders while mounted [40]. In relation to the present study, preloading, loading, and unloading activities are performed by handlers while unmounted. Estimates for the proportion of equine-related injuries sustained among populations of unmounted farm workers and equestrians range from 25 to 32% [40,41,42,43]. Values from occupational health studies focused on equine industry workers are as high as 84 to 96% [44,45]. Given the dearth of similarly focused studies to the one described in this manuscript [24,29], a comparison of the types of injuries described in the current study is made with other reports that encompass a range of unmounted activities not restricted to those associated with road transport. 

In the present study, injured respondents were associated with being female, younger, and less experienced. The median age of this group is consistent with that reported in a broader survey of hospital discharge and compensation claim data for horse-related sports and recreation injuries in New Zealand [41]. The survey’s authors also reported that more females than males were hospitalised, and they were younger than admitted males. However, most injuries were sustained while riding (98.2%; 703/713 cases) [41]. Australian hospitalised patient- or registry-based studies have found similar risk factors (i.e., female and late thirties) [9,40]. In contrast to this finding, studies comparing unmounted and mounted equestrians based on the University of Kentucky Trauma Registry and American College Surgeons National Trauma Bank found males more likely to be injured in an occupational setting or while unmounted in comparison with mounted equestrians, but there was no effect of age on injury occurrence [6,42]. There were more female nonoccupational injuries sustained while mounted. Regional differences among horse industry participant populations and their preferred equine discipline or activity may explain different demographic findings, as can differences in research methodology. For example, in Kentucky, the horse industry is dominated by thoroughbred breeding, with many male workers [45]. Caution should also be exercised when interpreting hospital- or registry-based data and relying upon them for estimates of risks because, as found in our study and others, many injuries are either not treated or medical assistance is not sought [46]. This behaviour may be a manifestation of the normalisation of risk in equestrian culture and/or the acceptance that horse-related activities such as riding are high risk, both of which have been considered obstacles to improving equine-related safety [2,14,38,47]. 

Injury types and severity are important in estimating the prognosis for owners and carers and assessing the associated economic and personal costs [6,9,40,41,42]. They also inform recommendations for improved equestrian and worker safety [1,2]. Bruising and soft tissue injuries, occurring singly or concurrently with other tissue damage, were most common and resulted from traumatic contact with the horse (i.e., a kick, bite, or being crushed) or inanimate structures [41]. This is also the most common injury sustained by animal science and veterinary students when engaged in unmounted learning activities (91.3%) [46] and among regional hospital patients (51%) in Australia [43]. More severe injuries were less common, in contrast with urban-hospital-based admissions studies, which tend to be biased towards those of more significant medical concern [40,42]. Of the more potentially life-threatening injuries, head trauma was uncommon (11%; 8/73) compared to hospital studies of unmounted equestrians [42] and markedly less common than the frequency reported for mounted equestrians [6,9,40,41,42]. Nevertheless, the severe consequences of head injury warrant using protective headwear when transporting horses, especially during loading and unloading. The use of personal protective equipment for horse owners and carers was not investigated in the current study, but it is well recognised that the occupational use of helmets is lower than for nonoccupational activities [6]. Rope burns and hand injuries were common in the present study population, more so than in unmounted animal and veterinary science students [46], and are likely due to the behavioural problems that were frequently encountered in loading and unloading of the horse population reported by the authors’ companion study [18]. The use of hand protection is recommended during unmounted activities associated with horses, including the road transport of horses [46]. 

To identify the focus for a further prospective study to mitigate the risk of injury, multivariable analyses of factors associated with equine-transport-related injury were performed. The educational qualifications evaluated are unique to New Zealand. However, pony club and equestrian and racing qualifications have many similarities in New Zealand, Australia, and the United Kingdom based on a shared colonial history. The increased odds of injury associated with higher levels of qualification are most likely reflective of those more frequently engaged in industry activities and, therefore, at greater cumulative exposure to the risk in associated competitive equestrian activities or equine industry work environments [30,38]. The association of a decrease in risk of injury with driver experience (2% per year) was not found for equine injury in a companion paper [30]. It is unclear if more experienced drivers are less distracted or better at managing fatigue, therefore resulting in fewer self-injuries when transporting horses. However, driver fatigue and distraction are associated with increased accidents involving horses and livestock [46,48]. The association between reporting a horse injured during transport and self-injury was discussed above. 

This study shares several limitations common to survey-based studies [34]. The survey distribution method was not randomised, as there is no national database of horse owners in New Zealand, and techniques such as random number selection from a phone book are impractical at a national level [49]. The online distribution method selects respondents with internet access and may exclude those without. There was also an opportunity for selection bias by attracting respondents who are more highly experienced in equine road transport or with injury experiences. A general limitation of studies of this type is self-reporting; observational research may be required to gain insight into revealed human behaviours. The higher participation of women in online surveys has been observed in similar studies [50] but is most likely reflective of the high participation rates of women in equestrian sports and recreational riding in New Zealand [51]. These data are subject to recall bias; hence the authors restricted the survey of transport-related injury to two years before completion. The findings in this study are likely an underestimation of the risk of injury, as information on near misses was not elicited [23,25]. Finally, retrospective surveys of this type may provide information on the association between risk factors and human injury but do not prove a confirmation of causation or protection.

Notwithstanding these limitations, the survey design made it possible to address the authors’ aim and established that the road transport of horses poses a significant risk of injury to horse handlers, especially during loading and unloading.

## 5. Conclusions

Our findings support the development and adoption of strategies designed to eliminate equine injuries associated with the road transport of horses to reduce the risk of injury to their owners and carers. Education and training have been proposed as an approach to lowering the occurrence of equine-related injury, but in the current study, the relationship between this and injury prevention needs to be clarified. It is likely, though not proven in the present study, that those with a greater engagement with the industry as workers or recreationally have completed a course of related training and education. However, it is unclear if these programs specifically address risk reduction or the training of horses for transport. Based on this and other recently published works on the injury of horses during transport by the authors and others, a review of education strategies that address this shared “one welfare” risk in the industry is prudent [1,52,53]. Any approach to reducing the shared risk of human and equine injury is most likely facilitated by addressing issues of horse welfare [14], multidisciplinary approaches to understanding horse-related risk [11], and engagement of the principles of equitation science to improve the safety of horse–human interactions [54]. Horse and handler training, personal protective equipment such as helmets and gloves, and safety awareness strategies should be developed that are specific to the at-risk demographic and industry activity [1,43].

## Figures and Tables

**Table 1:** The types of injury described by 73/112 respondents reporting injuries sustained while transporting horses by road in New Zealand.

Transport Phase	n	n ^1^	Injury Types										
			Bruising/Soft Tissue Injury	Crush Injury	Dislocation Sprain or Strain	Dental Injury	Facial Injury	Fracture	Head Injury	Internal Injury	Laceration/Open Wound	Muscle/Tendon Injury	Rope Burn
Preloading or handling	13	7 (7 ^2^; 0 ^3^)	2 (2; 0)	-	1 (1; 0)	-	-	-	-	-	-	2 (2; 0)	2 (2; 0)
Loading	39	33 (20; 13)	12 (4; 8)	8 (3; 5)	2 (1; 1)	1 (0; 1)	3 (1; 2)	3 (3; 0)	2 (1; 1)	-	2 (0; 2)	5 (2; 3)	14 (5; 9)
Traveling	6	5 (3; 2 ^4^)	2 (1; 1)	1 (0; 1)	-	1 (0; 1)	2 (0; 2)	2 (1; 1)	2 (0; 2)	1 (0; 1)	1 (0; 1)	1 (1; 0)	
Unloading	33	28 (14; 14)	20 (11; 9)	4 (0; 4)	1 (0; 1)	-	2 (0; 2)	2 (0; 2)	4 (0; 4)	-	2 (0; 2)	4 (0; 4)	10 (3; 7)
Not stated	21	-	-	-	-	-	-	-	-	-	-	-	-
Total	112	73 (44; 29)	36 (18; 18)	13 (4; 9)	4 (2; 2)	2 (0; 2)	7 (1; 6)	7 (4; 3)	8 (1; 7)	1 (0; 1)	5 (0; 5)	12 (5; 7)	26 (10; 16)

^1^ Number of respondents who provided further details of their injuries; 40% had concurrent injuries. ^2^ Indicates the number of respondents where this type of injury occurred singly. ^3^ Indicates the number of respondents where this type of injury occurred concurrently with other types of tissue injury. ^4^ One respondent had a combination of head injury, facial injury, dental injury, fracture, crush injury, internal injury, laceration or open wound, bruising/soft tissue injury, dislocation/sprain/strain, and muscle/tendon injury.

**Table 2:** The anatomic distribution of injuries described by 72/112 respondents reporting injuries sustained while transporting horses by road in New Zealand.

Transport Phase	n	n ^1^	Anatomic Regions Affected									
			Head or Face	Neck	Back	Arm	Hand	Pelvis	Leg	Foot	Chest	Stomach or Abdomen
Preloading or handling	13	7 (7 ^2^; 0 ^3^)	-	-	-	-	4 (4; 0)	-	1 (1; 0)	2 (2; 0)	-	-
Loading	39	31 (19; 12)	5 (1; 4)	1 (0; 1)	4 (1; 3)	6 (2; 4)	19 (11; 8)	2 (1; 1)	2 (1; 1)	7 (3; 4)	1 (0; 1)	1 (0; 1)
Traveling	6	5 (3; 2)	2 (1; 1)	1 (0; 1)	1 (1; 1)	-	1 (0; 1)	-	1 (0; 1)	1 (0; 1)	2 (1; 1)	1 (0; 1)
Unloading	33	28 (17; 11)	4 (0; 4)	1 (0; 1)	2 (0; 2)	6 (1; 5)	9 (7; 2)	-	5 (2; 3)	10 (6; 4)	3 (0; 3)	2 (1; 1)
Not recorded	21	-	-	-	-	-	-	-	-	-	-	-
Total	112	72 (46; 25)	11 (2; 9)	3 (0; 3)	7 (2; 6)	12 (3; 9)	33 (22; 11)	2 (1; 1)	9 (4; 5)	18 (9; 9)	6 (1; 5)	4 (1; 3)

^1^ Number of respondents who provided further details of the location of injuries; 39.9% of people had concurrent regions affected. ^2^ Indicates the number of respondents where this injury location occurred singly. ^3^ Indicates the number of respondents where this location of injury occurred concurrently with other injury locations.

**Table 3:** Results of univariate logistic regression analyses of associations between human injury sustained during the road transport of horses, and survey respondent demographics, respondent driving and horse handling experience, industry sector, transport vehicle and travel frequency, horse injury, and transport-related behavioural problems.

Variable Name	Category	Est. ^1^	SE ^2^	OR ^3^	95% CI ^4^	*p* ^5^
Sex	Female	−0.66	0.80	0.52	0.13–3.46	0.006
	Male	Ref				
Age (years)		−0.03	0.007	0.97	0.96–0.99	<0.0001
Driving experience (years)		−0.03	0.007	0.97	0.96–0.97	0.0002
Driving license class	Car—learner/restricted	−0.39	0.57	0.68	0.23–2.21	<0.0001
	Heavy vehicle	−1.37	0.53	0.25	0.93–0.76	
	Car—full	Ref				
Horse industry sector	Breeding	−1.30	0.81	0.27	0.04–1.15	0.04
	Equestrian sport	0.07	0.40	1.08	0.51–2.55	
	Racing	−0.67	0.46	0.51	0.21–1.32	
	Recreational	−0.25	0.44	0.78	0.33–1.96	
	Other	0.12	0.49	1.13	0.44–3.05	
	Pony club	Ref				
Horse handling experience (years)		−0.01	0.007	0.99	0.98–1.00	0.10
Highest horse industry qualification	Equestrian Sports New Zealand	0.72	0.37	2.06	0.07–4.17	0.001
	New Zealand National Certificate	0.77	0.33	2.16	1.10–4.09	
	Horse racing industry qualification	0.036	0.33	1.04	0.53–1.94	
	Other equine-related qualification	0.01	0.44	1.01	0.40–2.27	
	No formal training	−0.58	0.27	0.56	0.44–0.94	
	New Zealand Pony Club	Ref.				
The vehicle used for horse transport	Angle float/trailer	−1.23	1.18	0.29	0.04–6.10	0.04
	Straight float/trailer	−0.88	1.13	0.42	0.60–8.24	
	Small truck (2–3 horses)	−0.39	1.14	0.67	0.10–13.44	
	Large truck (>3 horses)	−0.36	1.15	0.70	0.10–14.09	
	Commercial truck	Ref				
Frequency of horse transport	Daily	0.91	0.45	2.49	1.02–6.20	0.04
	3–5 times a week	0.62	0.38	1.86	0.92–4.10	
	Once a week	0.40	0.39	1.49	0.72–3.34	
	Every two weeks	0.51	0.40	1.66	0.77–3.79	
	Once a month	0.22	0.47	1.25	0.50–3.15	
	Less than monthly	Ref				
Owner reported a transport-related horse injury in the survey		1.01	0.22	2.75	1.78–4.20	<0.0001
Owner reported a transport-related horse behavioural problem in the survey		−1.00	0.86	0.37	0.8–2.65	0.16

^1^ Coefficient estimate. ^2^ Standard error. ^3^ Odds ratio. ^4^ 95% Confidence interval. ^5^ Wald test *p* value. Ref = reference group.

**Table 4:** Results of multivariate logistic regression analyses of associations between human injury sustained during the road transport of horses, industry-related training qualifications, driving experience, and reporting a transport-related equine injury or behavioural problem.

Variable Name	Category	Est. ^1^	SE ^2^	OR ^3^	95% CI ^4^	*p*^5^ │>z│	*p* ^6^
Intercept		−2.10	0.25			<0.0001	
Highest horse industry qualification	Equestrian Sports New Zealand	1.07	0.40	2.93	1.29–6.34	0.008	0.0007
	New Zealand National Certificate	0.94	0.36	2.57	1.24–5.19	0.009	
	Horse racing industry qualification	0.70	0.36	2.02	0.98–4.08	0.052	
	Other equine-related qualification	0.28	0.48	1.32	0.47–3.23	0.563	
	No formal training	−0.28	0.30	0.76	0.42–1.36	0.354	
	New Zealand Pony Club	Ref.					
Driving experience (years)		−0.02	0.008	0.98	0.96–0.99	0.002	0.0002
Owner reported a transport-related horse injury		0.85	0.24	2.35	1.44–3.76	0.0005	0.0007

^1^ Coefficient estimate. ^2^ Standard error. ^3^ Odds ratio. ^4^ 95% Confidence interval. ^5^ Wald test *p* value for category within variable. ^6^ Wald test *p* value for variable. Ref = reference group.

## Data Availability

Data available on request due to restrictions (human ethics approval).

## Supplementary Materials

The following supporting information can be downloaded at: https://www.mdpi.com/article/10.3390/ani13101594/s1, Table S1: New Zealand Horse Transport and Injury Survey; Table S2: Frequency table of variables explored for their possible associations with human injuries sustained while transporting horses by road in New Zealand.
