# Supplementary material for: Human Injuries Associated with the Transport of Horses by Road
Source: Animals (Basel). 2023 May 10;13(10):1594. doi: 10.3390/ani13101594 (PMC10215915; doi:10.3390/ani13101594)
Supplement: Supplementary file 1 [file animals-13-01594-s001.zip › Table S1 New_Zealand_Horse_Transport_and_Injury_Survey.pdf]

Table S1. New Zealand Horse Transport and Injury Survey

This study aims to examine the transportation of horses in New Zealand. It will look in to the types of horse transport vehicles used and into the injury to horses or people during horse transport. Any information provided is anonymous and not linked back to you. This project has been registered as a low risk project with the Massey University Human Ethics committee. By completing this survey you are consenting to participate in the study. If you would like more information please contact Chris Riley.

- ❍ Yes, I give my consent - continue to the survey
- ❍ No, I do not give my consent - leave the survey

What is your sex?

- ❍ Male
- ❍ Female
- ❍ Other

What is your age (years)?

What is the postcode of the town or place where your horse is kept?

What section of the horse industry or sport are you primarily involved in?

- ❍ Thoroughbred racing
- ❍ Standardbred racing
- ❍ Pony club
- ❍ Endurance
- ❍ Horse breeding
- ❍ Recreational riding
- ❍ Dressage
- ❍ Show jumping
- ❍ Eventing
- ❍ Reining
- ❍ Showing
- ❍ Polo
- ❍ Other (please specify) ____________________

Are you a registered member of one or more of these organisations? (tick all that apply)

- ❍ I am not a registered member of any horse related organisation
- ❍ New Zealand Thoroughbred Racing
- ❍ Harness Racing New Zealand
- ❍ Equestrian Sports New Zealand
- ❍ New Zealand Pony Club Association
- ❍ Other (please specify) ____________________

What is your relationship with the horse(s)?

- ❍ Mostly amateur - involved with horses as a hobby or for recreational purposes ◎ Mostly professional - involved with horses as part of my job

What do you do professionally with horses?

What amateur activities do you do with horses?

How many years of experience do you have with handling horses?

Do you have any qualifications for the equine industry? (tick all that apply)

- ❑ New Zealand Pony club certificate (please specify) ____________________
- ❑ Racing (please specify) ____________________
- ❑ Equestrian Sports New Zealand qualification (please specify) ____________________ ◎ New Zealand national certificate - horse related (please specify) ____________________
- ❑ Other (please specify) ____________________
- ❑ No, I do not have any specific horse-related qualifications

What type of driving licence do you hold? (tick all that apply)

- ❑ Learners
- ❑ Restricted
- ❑ Full
- ❑ Motorcycle
- ❑ Heavy vehicle licence (class) ____________________

How often do you transport your horse(s)?

- ❍ Daily
- ❍ 2 to 5 times a week
- ❍ Once weekly
- ❍ Fortnightly
- ❍ Monthly
- ❍ Less than once a month

What is the average kilometers traveled per trip when you have transported your horse(s) in the last year?

How do you usually transport your horse(s)?

- ❍ Small truck - 2 to 3 horses
- ❍ Large truck - more than 3 horses
- ❍ Gooseneck
- ❍ Float/trailer - angle load
- ❍ Float/trailer - straight load
- ❍ Use a commercial trucking company

In your horse transport vehicle what direction is the horse facing?

- ❍ Head facing or angled to the front
- ❍ Head facing or angled to the rear
- ❍ Horse free and unrestrained

How likely are you to assess the fitness of your horse(s) for travel before transporting it?

- ◎ 1 - never
- ❍ 2 - sometimes
- ❍ 3 - about half the time
- ❍ 4 - most of the time
- ❍ 5 - always

Does your horse have a behavioural problems associated with transport? (e.g. anxiety, flight response, fight response, kicking, rearing, refusal to load)

- ❍ Yes (please describe) ____________________
- ❍ No

Has your horse(s) had any injuries related to transportation within the last 2 years?

- ❍ Yes
- ❍ No

Have you experienced an injury to yourself while you were transporting a horse in the last 2 years?

- ❍ No - leave the survey
- ❍ Yes

Did this injury happen at the same time as a horse was injured during transportation related activities?

- ❍ Yes - the same time as the horse described in this survey
- ❍ Yes - the same time as a horse not described in this survey
- ❍ No

When did you sustain the injury?

- ❍ Pre-loading or handling
- ❍ Loading
- ❍ Travelling

❍ Unloading

Do you want to provide more information about your injury?

❍ No - leave the survey
❍ Yes

What type of injury did you sustained and what part of the body did you injure?

Where did the injury occur?

❑ 1 - Head or face
❑ 2 - Neck
❑ 3 - Back
❑ 4 - Arm
❑ 5 - Hand
❑ 6 - Pelvis
❑ 7 - Leg
❑ 8 - Foot
❑ 9 - Chest
❑ 10 - Stomach or abdomen

What treatment did you seek for injury?

❍ None
❍ Self-treated
❍ First aid
❍ Medical assistance
❍ Hospital admission
❍ Other (please specify) ____________________

Have you recovered from the injury?

❍ No
❍ Yes (please specify the time it took for recovery) ____________________
