# Supplementary material for: Human Injuries Associated with the Transport of Horses by Road
Source: Animals (Basel). 2023 May 10;13(10):1594. doi: 10.3390/ani13101594 (PMC10215915; doi:10.3390/ani13101594)
Supplement: Supplementary file 1 [file animals-13-01594-s001.zip › Table S2 Frequency table of variable associations with human injuries during equine transport.pdf]

Table S2. Frequency table of variables explored for their possible associations with human injuries sustained while transporting horses by road in New Zealand.

		**Injured**	**Uninjured**	**All**
**Variable name**	**Category**	**n(%)[1]**	**n(%)[1]**	**n**
Sex	Female	102(11.5)	788(88.5)	890
	Male	7(4.3)	157(95.7)	164
	All respondents	109(10.3)	945(89.7)	1054
Age (years)		111(10.4)	952 (89.6)	1063
Driving experience (years)		97(9.9)	880(90.1)	977
Driving licence class	Car - full	47(7.6)	569(92.4)	616
	Heavy vehicle	41(11.3)	321(88.7)	362
	Car - learner/restricted	17(25.5)	50(74.6)	67
	All respondents	105(10.0)	940(90.0)	1045
Horse industry sector	Breeding	2(3.6)	53(96.4)	55
	Equestrian sport	54(12.4)	364(83.5)	436
	Racing	16(7.0)	214(93.0)	230
	Recreational	20(10.6)	187(98.9)	189
	Other	12(13.5)	77(86.5)	89
	Pony club	8(12.1)	58(87.9)	66
	All respondents	112(10.5)	953(89.5)	1065
Industry involvement	Amateur	79(9.9)	715(90.1)	794
	Professional	33(12.2)	238(87.8)	271
	All respondents	112(10.5)	953(89.5)	1065
Horse handling experience (years)		109(10.3)	948(89.7)	1057
Highest horse industry qualification	Equestrian Sports New Zealand	12(20.3)	47(79.7)	59
	New Zealand National Certificate	16(21.1)	60(78.9)	76
	Racing industry qualification	15(11.4)	117(88.6)	132
	Other equine related qualification	7(11.1)	56(88.9)	63
	No formal training	27((6.5)	390(93.5)	417
	New Zealand Pony Club	35(11.0)	283(89.0)	318
	All respondents	112(10.5)	953(89.5)	1065
The vehicle used for horse transport	Commercial truck	4(5.7)	66(94.3)	70
	Angle float/trailer	8(6.8)	109(93.2)	117
	Straight float/trailer	52(9.4)	499(90.6)	551
	Small truck (2-3 horses)	29(14.4)	172(85.6)	201
	Large truck (>3 horses)	18(14.9)	103(85.1)	121
	All respondents	111(10.5)	949(89.5)	1060
Frequency of checking the horse for fitness before travel	Never	1(5.9)	16(94.1)	17
	Sometimes	8(12.5)	56(87.5)	64
	Half the time	7(14.9)	40(85.1)	47
	Most times	28(11.6)	214(88.4)	242
	Always	67(9.7)	626(90.3)	693

	All respondents	111(10.4)	952(89.6)	1063
Frequency of horse transport	Daily	12(15.6)	65(84.4)	77
	3-5 times a week	33(12.1)	239(87.9)	272
	Once a week	26(10.0)	235(90.0)	261
	Every two weeks	21(10.9)	171(89.1)	192
	Once a month	10(8.5)	108(91.5)	118
	Less than monthly	10(6.9)	135(93.1)	145
	All respondents	112(10.5)	953(89.5)	1065
Owner reported a transport-related horse injury in the survey	Yes	38(20.2)	150(79.8)	188
	No	74(8.4)	803(91.6)	877
	All respondents	112(10.5)	953(89.5)	1065
Owner reported a transport-related horse behavioural problem in the survey	Yes	31(12.9)	210(87.1)	241
	No	79(9.7)	738(90.3)	817
	All respondents	110(10.4)	948(89.6)	1058

[1]Percentage of row total for all respondents that provided information in response to each question.
